# Pastoralist Community's Perception of Tuberculosis: A Quantitative Study from Shinille Area of Ethiopia

**DOI:** 10.1155/2013/475605

**Published:** 2013-11-27

**Authors:** Samuel Melaku, Hardeep Rai Sharma, Getahun Asres Alemie

**Affiliations:** ^1^Department of Public Health Nursing, Jijiga Health Science College, P.O. Box 504, Jijiga, Ethiopia; ^2^Institute of Environmental Studies, Kurukshetra University, Kurukshetra, Haryana 136119, India; ^3^Institute of Public Health, University of Gondar, P.O. Box 196, Gondar, Ethiopia

## Abstract

*Background*. In Ethiopia the prevalence of all forms of TB is estimated at 261/100 000 population, leading to an annual mortality rate of 64/100 000 population. The incidence rate of smear-positive TB is 108/100 000 population. *Objectives*. To assess knowledge, attitudes, and practices regarding TB among pastoralists in Shinille district, Somali region, Ethiopia. *Method*. A community-based cross-sectional study was conducted among 821 pastoralists aged >18 years and above from February to May, 2011 using self-structured questionnaire. *Results*. Most (92.8%) of the study participants heard about TB, but only 10.1% knew about its causative agent. Weight loss as main symptom, transmittance through respiratory air droplets, and sputum examination for diagnosis were the answers of 34.3%, 29.9%, and 37.9% of pastoralists, respectively. The majority (98.3%) of respondents reported that TB could be cured, of which 93.3% believed with modern drugs. About 41.3% of participants mentioned covering the nose and mouth during sneezing and coughing as a preventive measure. The multivariate logistic regression analysis indicated that household income >300 Ethiopian Birr and Somali ethnicity were associated with high TB knowledge. Regarding health seeking behaviour practice only 48.0% of the respondents preferred to visit government hospital and discuss their problems with doctors/health care providers. *Conclusion*. This study observed familiarity with gaps and low overall knowledge on TB and revealed negative attitudes like discrimination intentions in the studied pastoral community.

## 1. Introduction

Tuberculosis continues to be one of the most important global public health threats. About one-third of the world's population is estimated to be infected with tubercle *bacilli* and hence at risk of developing active disease. TB is a leading killer of people with HIV. According to the World Health Organization Global TB Report [1], in 2010 there were approximately 8.8 million incident cases of TB globally, of which 1.1 million were among people living with HIV. The disease has been recognized as major public health problem in Ethiopia including Somali regional state and in 2010 there were estimated 220,000 (261/100,000) incident cases of TB, estimated 29,000 deaths (35/100,000) excluding HIV-related mortality [1]. As per Federal Ministry of Health (FMoH) data, TB in Ethiopia was one of the leading causes of morbidity, the fourth main cause of hospital admission, and the second largest cause of hospital deaths (after malaria) [2]. According to the Somali Regional Health Bureau Report [3], the annual incidence rate during 2008 of smear-positive PTB was 175−250/100,000/year which was higher than the national figure of 165/100,000. The disease is one of the top ten causes of outpatient visit, hospital admission and death in the region. Furthermore, the emergence of multidrug resistant TB (MDR-TB) has become a major public health problem in a number of countries and an obstacle to global TB control [4, 5]. Ethiopia is one of the 27 high burden MDR-TB countries ranking 15th with more than 5000 estimated MDR-TB patients annually [2].

Pastoralists are the seminomadic people whose livelihood largely depends on livestock raising. An estimated 50–100 million pastoralists live in the developing world of which 60% are in sub-Saharan Africa. In the Horn of Africa, pastoralists constitute 70% of the general population, of which 12% is in Ethiopia [6]. The majority of these pastoralists live in the southeastern and northeastern Somali region of the country having three different livelihood systems, that is, pastoralism, agropastoralism, and urban. Pastoralists and agro-pastoralists both constitute about 85%, while the rest was urban. Due to their mobile lifestyle, pastoralists often stay in border areas and highly volatile and insecure environments that were often beyond the reach of formal health services including TB control programs [7]. Pastoralists in Shinille district of Somali region of Ethiopia were confined to the most arid part of the country. Being geographically isolated in remote rural areas with poor infrastructure and communication, they are considered to be underserved and deprived of all forms of health care and are perceived as of low priority. Work environment, poverty, and lack of awareness are important factors affecting the health seeking behavior and the control strategies. Previously, a study was conducted in the area to explore barriers delaying TB diagnosis among pastoralist TB patients. The study revealed that factors related to sociocultural, perceptions of TB, and pastoralists' limited access to health care are the key factors leading to an apparent delay in diagnosis of pastoralist TB patients [7]. Tuberculosis is still a problem in the area and further high notification rate poses a challenge. Since the perception of pastoralist community in the region, particularly in Shinille district, towards TB has not been well investigated, assessing the baseline information regarding knowledge, attitudes, and practices on TB will offer a good insight about the overall picture of the control activity in the region and thereby assist in the development of a strategy for improving quality of service.

## 2. Methods

Somali region (state) is the eastern-most of the nine ethnic divisions of Ethiopia. The capital of Somali State is Jijiga. Shinille, the largest of four districts in Shinille Zone which is located in the Somali Region, has a latitude and longitude of 09°41′N and 41°51′E with an elevation of 1079 meters above sea level. The region borders are Kenya to the south-west, the Ethiopian regions of Oromia, Somali, and Dire Dawa to the west, Djibouti to the north, and Somalia to the north, east, and south. The region is among the areas of the country sparsely populated with an average population density of about 15 persons per km^2^. The region is remote with a mobile nomadic population and inadequate infrastructure. Climatically, it is mostly desert with high average temperatures and low bimodal rainfall. Its economy is weak and reliant predominantly on traditional animal husbandry and marginal farming practices. Shinille district has an estimated total population of 13,132, of whom 6,758 were males and 6,374 were females according to Central Statistical Agency [8]. The two largest ethnic groups reported in the district were the Somali (96.58%) and the Oromo (1.76%), while all other ethnic groups made up the remaining 1.66% of the residents [8]. The major type of activity in population is pastoralism (48%) followed by crop farming (25%) and agropastoralism (17%).

A community-based cross-sectional survey was carried out from February to May 2011. The study population was the heads of pastoralist households with age above 18 years and residing in the study area. As per custom the heads of the family interact first with the society, community, and other visitors and are assumed to have more exposure and knowledge about social and health issues. Household heads having sickness, speaking and hearing problems were exempted from the study. In the absence of the family heads, the other eldest male or female member of the family was selected for the interview. The required sample of 852 household heads was determined using sample size calculation formula for single population proportion [9], with 95% confidence level, and 49.1% assumed prevalence of overall knowledge on TB according to a similar study done on pulmonary tuberculosis (PTB) in Arbaminch area of Ethiopia [10]. The study subjects were selected using cluster sampling technique. For cluster sampling, a design effect of 2 was applied and 10% was added for nonresponse rate accordingly for total sample size calculation. Shinille district has 28 villages and each village is considered as one cluster making a total of 28 clustered villages. Villages were selected randomly to represent the whole population and all households within the selected clusters were included in the sampling. Finally, from each housing unit the household head was selected for the interview. Pastoralists in the region are known to be ethnically and culturally a closely homogeneous group of people, who share one language, religion, and lifestyle. For such a closely homogeneous people by using cluster sampling technique, 852 household heads were deemed an adequate sample size to create the intended quantitative product. In addition because of scattered settlement of the communities on the vast areas of the region it can be expensive to survey. Treating several respondents within a local area as a cluster, finally all participants in the cluster were selected for Interview.

The data were collected by face-to-face interviews using self-structured and pretested questionnaires. The questionnaires were prepared by consulting the literature and modified to fit the local context. The questionnaire was first developed in English and then translated into Somali (local language), and back to English by a different individual to check consistency and conceptual equivalence. Included in the questionnaire were sociodemographic questions (age, sex, education, marital status, occupation, family size, monthly family income, and housing conditions), knowledge on TB causes, symptoms, diagnostic methods, transmission, prevention, treatment, and seriousness of disease, attitudes and practices intended health seeking behaviour, and information sources. Eight trained data collectors and one supervisor having good command on local language were involved in data collection. Prior to data collection, the objective of the study was discussed including practical exercise with data collectors and health education was provided regarding precautionary measures during data collection. An interview guide was also provided to data collectors and supervision was made by one of the authors during data collection process for timely edition of the data and feedback.

One of the authors coordinated the data collection process and rechecked the filled questionnaires. Pretesting of the questionnaire was conducted in *Error woreda* (block) village. During the pretest, the questionnaire was assessed for its clarity, understandability, completeness, reliability, as well as sensitivity of the subject matter. Corrections were made for difficulties and interview time was determined for completing each questionnaire.

The ethical clearance was obtained from the Institutional Review Board of the University of Gondar. Permission was also obtained from Somali Regional State and from administrative bodies of the district including *kebeles* (wards). Verbal consent was obtained from each respondent after explaining the confidentiality and voluntary participation features of the study. Moreover, the study questionnaire was anonymous and interview was conducted in a private setting to maintain privacy of the respondents for sensitive questions. Objectives of the study were explained to the respondents prior to the administration of the interview and confidentiality was maintained by omitting name of the respondents.

Complete data was entered and analyzed using Statistical Package of Social Sciences (SPSS) version 16 for windows statistical program. Results were summarized using descriptive statistics and presented by frequency tables, percentage, and charts. Association was computed between knowledge and sociodemographic variables using bivariate and multivariate analysis techniques. Logistic regression was done to assess the associations of factors with tuberculosis knowledge. The significance of associations are presented using *P* values and the 95% confidence interval of the adjusted odds ratios (AOR).

Overall, knowledge on TB was assessed using questions such as causes of TB, symptoms, diagnostic methods, transmission, prevention, treatment, and seriousness of disease. The responses to these questions were added together and scored for every study participant; the composite score was dichotomized using mean as a cut-off value. A score of one was assigned to correct responses, nil for incorrect/do not know answers. Afterwards, scores above the mean value were categorized under high knowledge and those below the mean value were labelled as having low knowledge on TB. The mean score was taken as the cut-off value and those scored above were categorised in having high TB knowledge. The mean and the median scores were 5.42 and 5.51, respectively. Similarly, attitudes, practices, and intended health seeking behaviour were assessed. Respondents attitude was measured by questionnaire containing two options of agree and disagree. Questions include *were they feel ashamed if some of their relative/family member gets infected with TB, want to keep secrete if they have TB, afraid of TB patients due to their illness, having belief that getting TB is a punishment for sinful act, continue friendship if their friends has TB, willing to provide care to their relative suffering from TB, might allow their daughter/son to marry cured TB patient*.

## 3. Results and Discussion

A total of 852 respondents aged ≥18 years were selected for this study. Out of this, 2.8% refused to participate while 0.82% could not be available for the interview after two attempts. Lack of interest was the reason for those who refused to participate. Therefore, the data was collected from 821 study subjects out of whom 51.6% were males and 48.4% were females with a response rate of 96.36%.

The highest proportion (30.1%) of the study subjects was within the age group of 25–34 years, and the least proportion (3.5%) was ≥55 years. Majority of the respondents were married (81.0%), while 13.2% were single, and 2.3%, 2.1%, and 1.5% were widowed, separated, and divorced, respectively. More than half of the households (55.2%) had a mean family size of 5. Regarding the occupation of the interviewee, pastoralists were dominant (43.8%), followed by cattle keepers and farmers (34.2%), agro-pastoralists (20.1%), and the rest (1.8%) included merchants, Quran tutors, and shop keepers (Table 1). Regarding pastoralists' education, 62% were illiterate, that is, cannot read and write. Around 34% can read and write, while 3.9% and 0.2% had 1–8 and 9–12 standard education, respectively. All of the study subjects were Muslims and a substantial amount (94.5%) identified themselves with the Somali Ethnic group and the rest (5.5%) with Oromo ethnicity. The median monthly income of the families was between 100 and 300 Birr (Ethiopian currency) approximately equivalent to 5.88–17.65 US$.

The study demonstrated that TB was familiar among pastoralist communities in the study area. The majority (92.8%) of the participants (52.2% males and 47.8% females) had heard about TB (*Kahoo* in the local language), similar to the results from other Ethiopian studies [10, 11]. The common sources of information mentioned by the respondents were radio (38.6%), health service providers (33.2%), and the rest from friends, schools, and relatives (Table 2). As newspapers and televisions were not commonly used in the study area, most of the Somalis had radio listening habit especially the Somali BBC which could be the main source of health information including TB, similar to the study carried out in Nepal [12].

Even though TB is familiar in the study area, there is a wide knowledge gap regarding the causes of the disease among the respondents. Only 10.1% knew bacteria as the medical cause of TB, whereas the majority of the respondents (89.9%) did not know about the cause. The perceived TB causes among 89.9% pastoralists varied from animals to cold air, food shortage, smoking, chewing khat leaves (*Catha edulis forsk*), poverty, hand shaking, sexual intercourse, and even magic (Table 3). Dualeh [13] reported internal injury due to hard work and malnutrition as additional perceived causes of TB. As also documented in other Ethiopian studies, low knowledge regarding medical cause of TB might be due to low education coverage or due to its geographical landscape where many rural and mountainous areas were difficult to access by the health extension workers [11, 14]. Being a pastoralist, and because of the need for child labour, has let the school enrolment rates very low in Somali region having an adult literacy rate for men and women of 15% and 12%, respectively [15]. As limited geographical areas are given to HEWs and accessibility is granted through existing monitoring system, however, due to high mobile nature of the community the accessibility and use of health benefits schemes are always doubtful. Lack of knowledge delays in healthcare seeking behavior, diagnosis and treatment, resulted in further increase transmission rate, morbidity, mortality, and socioeconomic problems. About 11% and 2.5% of the participants, respectively, believe in hand shaking and sexual intercourse as TB transmission mode (Table 3) and about 4% respondents avoid hand shaking to prevent TB (Table 5). The community misconception regarding hand shaking and sexual intercourse as TB transmission mode may favor wrong attitude development in the society for patients.

**Figure 1:**
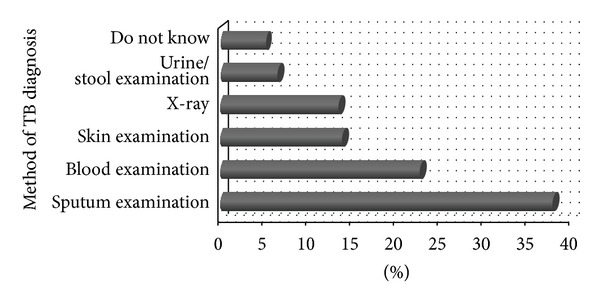
Percentage distribution of respondents by knowledge on method of TB diagnosis.

Regarding the best method for TB diagnosis, the present study revealed that 37.9% of the participants reported correct sputum examination while the rest (62.1%) mentioned blood diagnosis, skin and urine and stool examination, and X-ray methods (Figure 1). Most (63.7%) of the study participants in Nepal reported sputum examination as the main TB infection diagnostic method while others mentioned blood and skin examination, physical examination, and X-ray [12].

The studied pastoralists had good knowledge on TB prevention. About 41.3% expressed nose and mouth covering during sneezing and coughing and 21.0% stated BCG vaccination as preventive methods. Regarding disease treatment, majority of the Shinille pastoralists (98.3%) knew that TB is curable (Table 4) which is in accordance with the study carried out in Afar district, Ethiopia [10]. Majority of the study participants (93.3%) believed that TB treatment is well done with modern medicines. In earlier study from Ethiopia 87.7% informants reported the use of modern drugs as a better option, whereas the rest mentioned both modern drugs and traditional medicines [10].

In accordance with finding from Gondar city of Ethiopia [16], 47.4% of the study participants reported that anybody can be infected with TB while others reported poor, homeless people, alcoholics, drug users, people living with HIV/AIDS, and prisoners could be infected (Table 5). Majority (93.7%) of the pastoralists believed animals to be the source of TB, and when asked about that, 30.1% responded about sharing same shelter with animals, 25.8% replied about drinking water from the common source and drinking raw milk (18.3%), eating raw meat (13.3%), and drinking raw animal blood (12.5%). Regarding housing conditions and TB, majority (95.3%) of the respondents believed that TB had relation with housing conditions like cooking and sleeping in same room (36.0%), improper ventilation (31.0%), and house cleanliness (33%). Smoking and alcohol consumption have also been cited in other studies conducted in India and Kenya [17, 18]. Respondents' perception of overcrowding of people sleeping in windowless rooms as a risk factor in acquiring infections among the pastoralists in Arusha, Tanzania [19]. In Eastern Ethiopia living in a single room was one of the reported factors that increased the risk of acquiring TB infection [11]. Hence, attention should be given to prevent disease transmission from lack of ventilation since in the study area all family members culturally construct single room house without windows and used it for cooking and sleeping purposes.

The present study showed high proportion of negative attitudes among pastoralist community. The common attitude found was feeling ashamed if relative/family member gets infected with TB (62.2%), keeping it secret if self or family member/relative get TB infection (43.4%), being afraid of TB patients (31.1%), considering the disease as a punishment for sinful act (34.8%), and believing that TB affects breast feeding (46.2%). Almost half of the study participants (49.9%) were not willing to provide care to their relatives suffering with TB, do not allow their children to marry cured TB patient (67.2%), a man/woman once having TB becomes infertile (36%), and around 26.6% of the informants were not willing to perform religious ceremonies with TB patients on treatment. Some respondents in Andhra Pradesh state of India attributed TB disease to sin, wrath of deities, witchcraft, evil eye, fate, and so forth [20]. In a study in Philippines respondents described TB as being shameful and a “*bad mark on the family*” [21].

**Figure 2:**
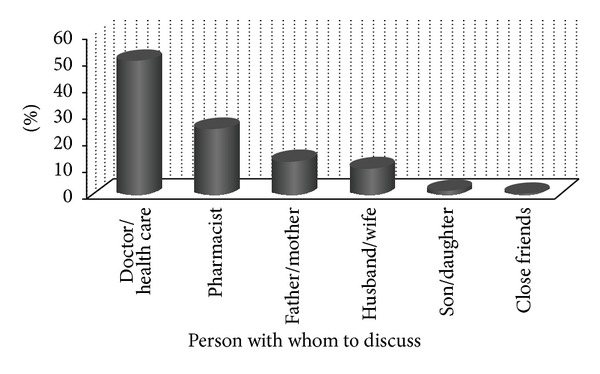
Percentage distribution of respondents to discuss their problem on TB infection.

**Figure 3:**
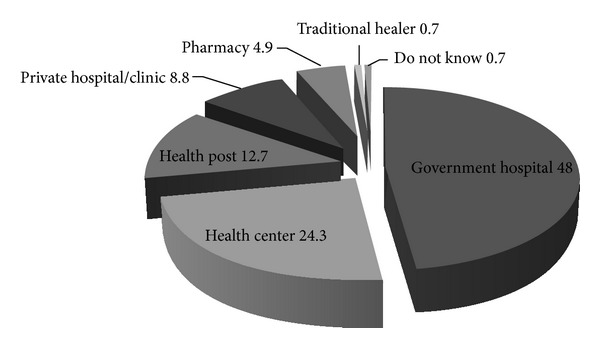
Percentage distribution of respondents preference for TB treatment.

The current study revealed that about half of the study participants (50.3%) discuss their problem with doctors/health care provider, while other discussed with pharmacist, parents, spouse, children, and close friends accordingly (Figure 2). The health seeking behaviour revealed that 48.0% and 24.3% of the respondents, respectively, preferred to visit government hospital and health centre if affected with TB (Figure 3) which might be attributed to the current TB control due strategy of Ethiopia where the FMoH creates awareness about TB through regular advertisement using mass media. The media also advises and encourages TB infected individuals to visit nearby government and private health institution and discuss their problems with health care providers. However, the percentage of TB affected people visiting hospitals is quite low when compared with that of 74.3% reported from Nepal [12].

Regarding the pastoralist daily activities to avoid TB, 22.3% of them kept proper room ventilation, 15.0% do not sleep in the same room with animals, 14.6% do not share bed with sick patients, 13.9% do not drink raw milk, 12.2% do not eat raw meat, and do not share utensils with sick patients (11.0%) (Table 5). The present study found that about 99.5% of the respondents practised hand washing especially after collecting dung and touching the sick animals which is an encouraging fact which must be sustained among the society.

The current study revealed that, out of all sociodemographic variables tested, occupation, family size, and household income/month had significance association and become a predictive of overall high TB knowledge. Results from multivariable logistic regression analysis (Table 6) showed that agro-pastoralist, as an occupation, is less likely to be predictive of overall knowledge of TB at (AOR: 1.21, 95% CI: (0.73–2.02)) than pastoralists, which is consistent with the finding of a previous study from Eastern Ethiopia [7] and in pastoral and agro-pastoral communities in Tanzania [19]. This might be because nomadic pastoralists have least access to health and other social services [22, 23]. This study reported that the participants whose household income >300 Birr/month (AOR: 2.03, 95% CI: (1.06–3.86)) were associated with having high knowledge on TB compared to those who had income <300 Birr/month; this might be explained as income increased the chance of getting access to information, education and seeking health care also increased. This study also found that pastoralists having family size of <five become a predictive of high overall knowledge of TB than those having family size >five at (AOR: 0.05, 95% CI: (0.04–0.08)).

## 4. Conclusion

This study documented familiarity towards TB but observed gaps and low overall knowledge about the disease in the studied pastoral community. Negative attitudes especially discrimination with infected individuals were also revealed. The study participants' practices or activities in avoiding TB and their health seeking behaviour were interesting and should be appreciated by the regional health bureau as it is one of the control strategies for TB. It is vital, therefore, for the regional health bureau to find ways of improving pastoralist's knowledge gap on TB and design strategies to create positive attitude to avoid discrimination of infected individuals with the disease among pastoralist. The regional health bureau should strengthen the available mobile health workers and promote an extensive health education programme to raise the awareness specifically about TB symptoms, means of transmission, prevention, and treatment in relation to the community misconceptions. Training should also be provided to members of pastoralist communities including traditional healers regarding TB control programs. Further research is suggested among pastoralists and control groups and different livelihoods systems in the study area.

## Figures and Tables

**Table 1:** Sociodemographic characteristics of the study population (*n* = 821).

Background Characteristics	Number (%)
Sex	
Male	424 (51.6)
Female	397 (48.4)
Age (years)	
18–24	197 (24.0)
25–34	247 (30.1)
35–44	212 (25.8)
45–54	127 (15.5)
>55	29 (3.5)
Do not know	9 (1.1)
Ethnicity	
Somali	776 (94.5)
Oromo	45 (5.5)
Marital status	
Married	665 (81.0)
Single	108 (13.2)
Separated	12 (1.5)
Divorced	17 (2.1)
Widowed	19 (2.3)
Occupation	
Pastoralist	360 (43.8)
Agro pastoralist	165 (20.1)
Cattle keeping and farming	281 (34.2)
Others*	15 (1.8)
Education	
Cannot read and write	509 (62.0)
Read and write	278 (33.9)
Grade 1–8	32 (3.9)
Grade 9–12	02 (0. 2)
Religion	
Muslim	821 (100)
Household income	
Less than 100 Birr	243 (29.6)
100–300 Birr	424 (51.6)
>300 Birr	77 (9.4)
Refuse to disclose income	77 (9.4)

*merchants, Quran tutors, shop keepers.

**Table 2:** Distributions of respondents by source of information on TB (*n* = 821).

Variables	Frequency (%)
Heard of TB	
Yes	762 (92.8)
No	59 (7.20)
Source of information	
Radio	294 (38.6)
Health service provider	253 (33.2)
Friends/Relatives	163 (21.4)
Schools/Teachers	52 (6.8)

**Table 3:** Knowledge regarding causes, transmission sources, and TB prevention modes (*n* = 762).

Indicators of knowledge	Number (%)
Causes of Tuberculosis	
Cold air	213 (28)
Shortage of food	179 (23.5)
Smoking/chewing	99 (13)
Dust	87 (11.4)
Poverty	79 (10.3)
Bacteria/germ	77 (10.1)
Animals	22 (2.9)
Magic	05 (0.7)
Others*	01 (0.1)
Symptom of TB	
Weight loss	261 (34.3)
Coughing blood	154 (20.2)
Cough lasting >2 weeks	88 (11.5)
Chest pain	75 (9.8)
Vomiting	58 (7.6)
Weakness	57 (7.5)
Loss of appetite	35 (4.6)
Shortness of breath	26 (3.4)
Fever/sweating	02 (0.3)
Do not know	06 (0.8)
Mode of transmission	
By droplet through air	228 (29.9)
Eating utensils	180 (23.6)
Drinking raw milk	142 (18.6)
Hand shaking	87 (11.4)
Sharing towels	74 (9.7)
Sexual intercourse	19 (2.5)
Other**	10 (1.4)
Do not know	22 (2.9)

*Sinful act, **drinking raw animal blood and meat.

**Table 4:** Percentage distribution of respondents' knowledge regarding TB treatment (*n* = 762).

Variable	Frequency		Total
	Male	Female	
TB is treatable	No (%)	No (%)	No (%)
Yes	389 (51.0)	360 (47.2)	749 (98.3)
No	09 (1.2)	04 (0.5)	13 (1.7)
TB treatment better through			
Modern drug	369 (48.4)	342 (44.9)	711 (93.3)
Traditional	03 (0.4)	05 (0.7)	08 (1.1)
Both	19 (2.5)	11 (1.4)	30 (3.9)
Do not know	07 (0.9)	06 (0.8)	13 (1.7)

**Table 5:** Respondents' knowledge on transmission and prevention of TB.

Indicators of knowledge about TB transmission and prevention	Frequency
	Number (%)
Who can be infected with TB	
Anybody	361 (47.4)
Poor people	149 (19.6)
Homeless people	101 (13.3)
Alcoholics	65 (8.5)
Drug users	29 (3.8)
People living with HIV	19 (2.5)
Prisoners	04 (0.5)
Do not know	34 (4.4)
Preventive methods	
Covering nose and mouth	315 (41.3)
Through balanced diet	86 (11.3)
BCG vaccination	160 (21.1)
Avoid smoking	125 (16.1)
Avoid drinking alcohol	23 (3.3)
Avoid handshaking	31 (4.1)
Avoid sharing dishes	08 (1.0)
Not sharing bed with others	13 (1.7)
Others*	01 (0.1)

*Early treatment, food, and by avoiding sex.

**Table 6:** Crude and adjusted odds ratio (OR) and 95% confidence intervals (CI) of determinants of TB knowledge.

Predictors	Knowledgeable on TB		Crude OR (95% CI)	Adjusted OR (95% CI)
	High	Low		
Sex				
Male	157	207	1	1
Female*	191	207	0.82 (0.62−1.04)	0.69 (0.47−1.02)
Age (Years)				
18–24*	97	94	1	1
25–34	117	119	0.14 (0.17−1.15)	5.31 (0.33−86.13)
35–44	83	111	0.14 (0.18−1.20)	0.32 (0.09−1.13)
45–54	43	72	0.19 (0.23−1.58)	0.36 (0.11−1.26)
55+	7	11	0.24 (0.03−2.01)	0.50 (0.14−1.74)
Do not know	1	7	0.02 (0.02−2.23)	0.77 (0.21−2.80)
Marital status				
Married*	274	342	1	1
Unmarried	53	50	0.76 (0.49−1.15)	0.57 (0.17−1.94)
Separated	7	4	0.46 (0.13−1.58)	0.69 (0.19−2.51)
Divorced	9	6	0.53 (0.19−1.52)	0.29 (0.04−2.18)
Widowed	5	12	1.92 (0.66−5.52)	0.60 (0.09−3.93)
Occupation				
Pastoralist*	142	197	1	1
Agro pastoralist	62	93	1.08 (0.73−1.59)	1.21 (0.73−2.02)
Cattle keeping and farming	135	119	0.63 (0.46−0.88)	0.45 (0.29−0.69)
Others	9	5	0.40 (0.13−1.22)	0.21 (0.05−1.77)
Ethnicity				
Somali	324	397	1	1
Oromo*	24	17	0.57 (0.30−1.09)	2.24 (0.99−5.07)
Household income				
Less than 100 Birr*	103	122	1	1
100–300 Birr	178	220	1.69 (0.98−2.93)	1.86 (0.93−3.72)
>300 Birr	27	44	1.76 (1.05−2.97)	2.03 (1.06−3.86)
Refuse to answer	40	28	2.33 (1.18−4.59)	3.04 (1.29−7.18)
Family size				
≤five*	112	370	1	1
>five	236	44	0.05 (0.04−0.08)	0.05 (0.03−0.07)

Knowledge score 5.4 (maximum of 8 score) was used as a cut off for comparison.

Variables significantly related (*P* < .05) to knowledge score on univariate analysis were included for multivariate logistic regression analysis. *Reference category.
